# Hepatitis B Virus Molecular Epidemiology, Host-Virus Interaction, Coinfection, and Laboratory Diagnosis in the MENA Region: An Update

**DOI:** 10.3390/pathogens8020063

**Published:** 2019-05-11

**Authors:** Duaa W. Al-Sadeq, Sara A. Taleb, Roan E. Zaied, Sara M. Fahad, Maria K. Smatti, Balsam R. Rizeq, Asmaa A. Al Thani, Hadi M. Yassine, Gheyath K. Nasrallah

**Affiliations:** 1 Biomedical Research Center, Qatar University, Doha 2713, Qatar; da1206066@qu.edu.qa (D.W.A.-S.); 200452443@student.qu.edu.qa (S.M.F.); msmatti@qu.edu.qa (M.K.S.); brizeq@qu.edu.qa (B.R.R.); aaja@qu.edu.qa (A.A.A.T.); hyassine@qu.edu.qa (H.M.Y.); 2 Biomedical Science Department, College of Health Sciences, Qatar University, Doha 2713, Qatar; st1000813@student.qu.edu.qa (S.A.T.); rz1407001@student.qu.edu.qa (R.E.Z.); 3 Department of Biological and Environmental Sciences, College of Arts & Sciences, Qatar University, Doha 2713, Qatar

**Keywords:** HBV, seroprevalence, genotypes, pathogenesis, transfusion, viremia

## Abstract

Hepatitis B virus (HBV) is an enveloped partial double-stranded DNA virus that can cause acute and chronic hepatitis. According to the World Health Organization (WHO) and the Centers for Disease Control and Prevention (CDC), 257 million people are living with HBV. Moreover, 20,900 acute hepatitis B cases were reported in 2016. Hepatitis B is highly prevalent in the African, Western Pacific, Eastern Mediterranean, South-East Asia, and European regions, respectively. Due to the high mutational rate of HBV and lack of reverse transcriptase proofreading activity, ten different genotypes with different geographical distributions have been identified. HBV pathogenesis and severity of infection depend on several host and viral factors, particularly, the genetic variability of both the host and virus. Although HBV infection is a global health concern, there is a lack of adequate studies and reports in the Middle East and North Africa (MENA) region. Here, we provide a review on HBV epidemiology, pathogenesis, host–pathogen interactions, coinfection with selected viruses, and laboratory diagnosis, focusing on studies conducted in the MENA region to determine the current situation of the HBV infection and outline the future study areas.

## 1. Introduction

Hepatitis is a worldwide health problem resulting in liver malfunction [1]. Although the primary cause of hepatitis is viral infections including HBV, other non-viral causes such as toxins, drugs, autoimmune diseases, infections with bacteria, as well as parasites, can also lead to hepatitis [2]. HBV is a partially double-stranded DNA virus that belongs to the *Hepadnaviridae* family, in the *Orthohepadnavirus* genus [3]. It is the causative agent of hepatitis B infection, resulting in both acute and chronic hepatitis infections. Chronic HBV infection can progress to hepatocellular carcinoma (HCC) and liver cirrhosis and subsequently leads to death. Therefore, it is considered a life-threatening virus worldwide, leading to significant rates of mortality [4]. According to WHO, 257 million people are living with HBV infection with an estimated number of 887,000 deaths in 2015 attributed to HBV complications [4]. In the USA, there are more than two million people living with chronic HBV-infection [2]. According to WHO epidemiological map, HBV is classified as a highly endemic virus in the Western Pacific, sub-Saharan Africa, and in East Asia. In the Arabian Gulf region, many immigrant workers come from highly HBV endemic areas such as Africa and East Asia. Hence, it is expected that the prevalence of HBV in migrants from these countries would be high, considering the ethnic diversity of the population [4]. Indeed, studies conducted in the Arabian Gulf region reported HBV seroprevalence to be between 2–7% [5]. Factors that might also lead to a high prevalence of the HBV infection the MENA region will be discussed below. The prevalence of HBV infection in different countries in the MENA region according to hepatitis B surface antigen (HBsAg) marker is summarized in Table 1. A review of recent peer-reviewed literature was conducted in different databases such as PubMed, Science Direct, Web of Science, and Scopus. Our search strategy utilized different combinations of search terms, such as ‘pathogenesis’, ‘genotype’, OR ‘HBV’ together with the name of each Arab country as ‘affiliation’ or ‘title’. Articles were screened based on the title and abstract. Eligible articles were fully reviewed and screened for the sample size, assay used, and reported prevalence for all Arab countries.

Direct exposure to infected body fluids or blood, unprotected sex, and vertical transmission are the main routes of HBV transmission [4]. The vertical transmission from an infected mother to her baby is mainly perinatal. Other routes of viral transmission could be through the shared use of non-sterile needles, toothbrushes, razors, or medical equipment contaminated with infected blood. Although HBV is a transfusion-transmissible virus, the risk of transmission through blood transfusions decreased decades ago due to the implementation of strict safety measures. The symptoms of HBV are not distinguishable from other hepatitis infections [4]. In addition, many people may not show any symptoms or even undergo unnoticed (silent) acute or chronic infection. Despite that, Hepatitis B has different infection stages characterized by the presence of specific biomarkers such as viral antigens, antibodies against different viral antigens, or viral DNA. Therefore, HBV infection can be detected serologically by either screening for HBsAg and hepatitis B envelope antigen (HBeAg), or screening for antibodies against the core protein (anti-HBc) or e antigen (anti-HBe). Most importantly, HBV DNA and viral load can be detected by polymerase chain reaction (PCR) [2].

This review aims to provide insights into HBV by reviewing recent reports covering HBV genotypes epidemiology, coinfection with other viruses, serological and molecular detection, in addition to the viral genetics and host factors associated with HBV pathogenesis. Special attention will be given to the studies conducted in the Arabian Gulf area and MENA regions.

## 2. HBV Genotype Distribution and Clinical Relevance

**Figure 1:**
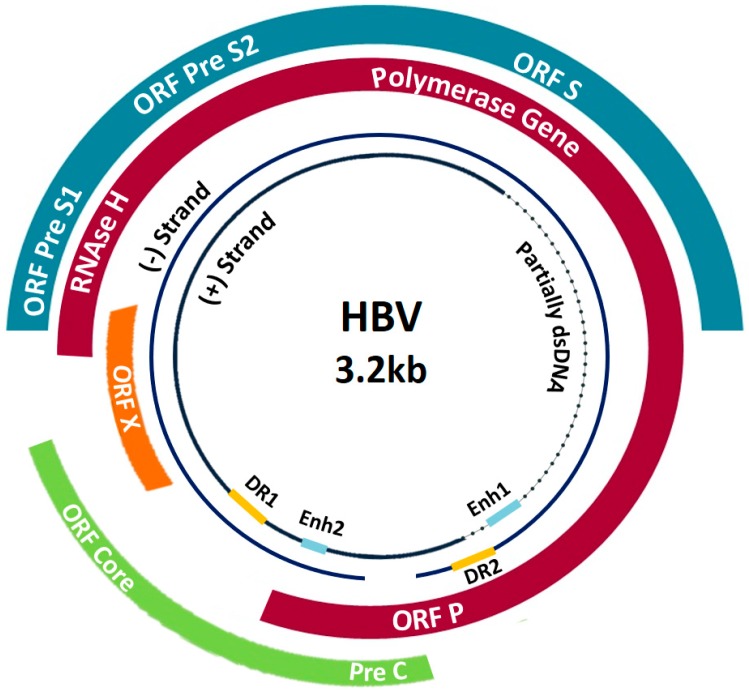
Schematic representation of the HBV genome. The genome is approximately 3020 nucleotides long and consists of partially double-stranded DNA. There are four overlapping open reading frames, four promoters, and two enhancer elements to regulate the transcription of viral RNA. The gene S encodes for the HBsAg, and it is a long open reading frame containing three start codons. Thus, the gene is divided into three sections, pre-S1, pre-S2, and S. The core gene consists of the pre-core and core regions, which encode for the HBV e antigen (HBeAg) and core protein, respectively. The polymerase (P) gene overlaps the entire S gene and encodes the viral DNA polymerase. Hepatitis B x antigen (HBxAg) is the smallest gene and is associated with the activation of transcription. The negative-sense strand is complementary to the viral mRNA. Using covalently closed circular DNA (cccDNA) as a template; the viral genes are transcribed by the cellular RNA polymerase II in the nucleus. DR1 and DR2 are 11-base-pair direct repeats that are required for strand-specific DNA synthesis during the HBV replication. Two enhancers (Enh1 and Enh2) exhibit activity in regulating the expression of the complete viral transcripts.

**Figure 2:**
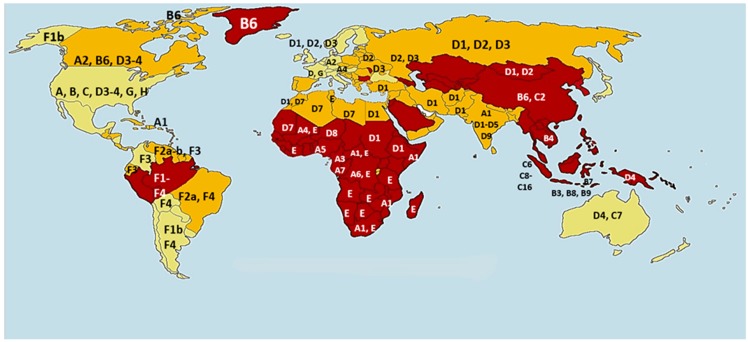
Worldwide geographic distribution of HBV genotypes. HBV has ten established genotypes that have different global and epidemiological distribution. Red color represents countries with high HBV prevalence, orange represents countries with moderate HBV prevalence, and yellow are countries with low HBV prevalence. Letters represent genotype and sub-genotype prevalent in each country.

In the Arabian Gulf region, the average prevalence of HBV-infection is reported to be between 2–7%. However, in some countries can reach up to 16.9% and 21.3% such as in Yemen and Sudan, respectively (Table 1) [5,50]. HBV is the second most common causative agent for HCC in most MENA countries following hepatitis C virus (HCV) [51,52]. The high prevalence of the HBV infection in the MENA region could be due to several factors (discussed below) including its genetic variability and heterogeneity. The genetic variability of HBV might be explained by its genome structure, which harbors a reverse transcriptase that facilitates the replication of the viral genomic DNA (Figure 1), due to the lack of proofreading mechanism of the HBV reverse transcriptase and high viral replication rate, which is around 1.4–3.6 × 10^−5^ substitutions per nucleotide site per cell infection [53]. HBV mutation rates are 100 times higher than other DNA viruses (ranging from DNA 10^−8^ to 10^−6^) and almost similar to RNA viruses (ranging from 10^−6^ and 10^−4^) [54,55]. For instance, Peck et al. concluded from their study that HCV and HIV has almost similar mutation rate as HBV. In addition, due to high immigration rate in the Arabian Gulf region from different countries in the world, including those of highly endemic region, this might lead to coinfection with different HBV genotypes and generation of new hybrid genotypes. In the presence of selective pressure against HBV, caused by immune responses or antiviral therapies, mutant and hybrid species can survive and dominate. To date, many genotypes and subtypes have emerged (Figure 2). There are 10 HBV genotypes, distributed across different geographical regions worldwide (all reviewed in-details in [3]). For instance, genotype A, B, and C are common in the Asian continent, and viral mutations were frequently associated with genotype C [56]. Genotype A is also highly prevalent in Europe and Africa, while West Africa is known for a restricted genotype, which is E. On the other hand, genotype D was found to be the most prevalent genotype in the Middle East. HBV genotypes are distinguished by a divergence in the nucleotide sequence of 8%, while subtypes under each genotype have a divergence of 4% [57].

It is believed that genotyping of HBV is important since HBV genotypes differ in terms of disease severity and pathogenicity response to interferon treatment. For instance, liver cirrhosis and progression to liver cancer are more commonly associated with HBV genotypes C and D compared to other genotypes [56]. However, genotype H, which is found in Central America, has lower pathogenicity in comparison to other genotypes. The low pathogenicity of genotype H could be attributed to the low viral replication rate and the altered expression of envelope proteins compared to genotype D as suggested by Sozzi et al. [58]. Moreover, a study aimed to investigate the epidemiological distribution as well as the correlation between clinical outcomes and genotypes, showed that genotype D was the most common genotype in Saudi Arabia, followed by genotype A, then E. Surprisingly, HBeAg levels were significantly lower in genotype D patients’ sera in comparison to those of genotype A and E patients, suggesting that genotype D has a better survival and immunoevasion mechanisms than other genotypes independent from high viral replication. These findings contradict Sozzi et al. who draw their conclusion based on observations from in vitro infection experiments. In regard to progression to liver cancer, the association between HBV genotype D infection and developing HCC has been wildly investigated in Egypt [59,60]. A group of researchers investigated genotype D through sub-typing and partial sequencing of preS1/preS2 DNA region to examine the impact of genetic variability of this region on HCC development [60]. The study concluded that HCC was not relevant to genotype D nor preS1/preS2 mutations.

Genotype D was found to be associated with occult hepatitis B virus infection (OBI) in the MENA region. It is a condition in which the level of HBsAg is undetectable with low levels of serum and/or liver HBV DNA [61]. In Saudi Arabia, it was found that OBIs prevalence reached 0.2% among blood donors and genotype D was predominant [37,62]. Although screening for OBI relies on HBc antibody, this study recommended screening blood donors using sensitive molecular testing in addition to HBc antibodies to exclude all cases of OBI. Another study conducted in Egypt has also shown similar findings in which OBI was associated with genotype D more than other genotypes [63]. Moving to Iran, HBV has been studied extensively, and several studies agreed on the predominance of genotype D [63,64]. Similarly, in Oman where there is an intermediate prevalence of HBV, a study by Al Baqlani et al. showed that genotypes D and A are predominant, followed by C and E, which were less prevalent, represented by a minority of the cases. In addition, the later study highlighted the importance of sequencing to detect mutations especially in case of antiviral treatment resistance [65]. However, although a similar study conducted on the Palestinian population also showed that genotype D was predominating, yet, this study also showed that genetic variation and mutations in HBV polymerase gene were unlikely to cause treatment resistance [66]. In summary, it is noticeable that genotype D is the most common genotype found in the MENA region with few exceptions. For instance, a study conducted in Egypt highlighted that HBV genotypes genotype E, but not D, was the most prevalent among healthcare workers [67]. Although there is some evidence, the relationship between genetic variability and disease progression in the MENA region needs further investigation. However, it seems that genotype D causes the most severe form of HBV viral hepatitis in the MENA region.

## 3. HBV Pathogenesis Associated with Host-Virus Interactions

### 3.1. Host Factors: Genetic Variations

**Figure 3:**
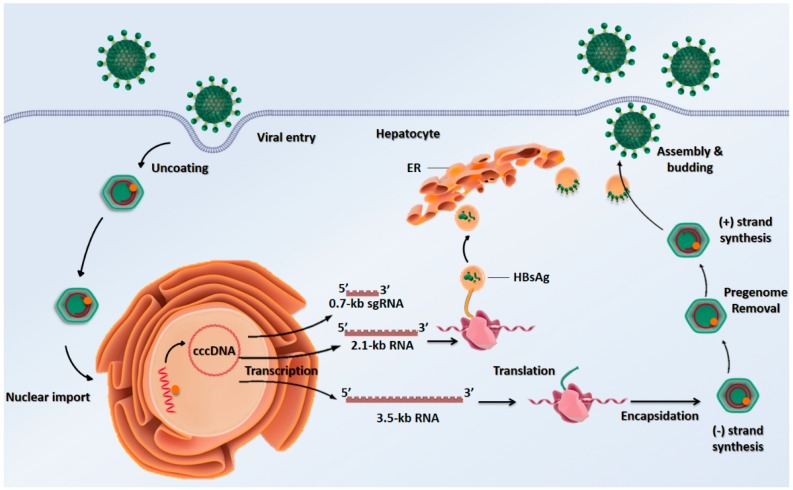
Schematic representation of the HBV life cycle. HBV attaches to the host hepatocyte cell membrane through its envelope proteins. When the viral membrane fuses with the cell membrane, it will result in releasing the viral genome into the cell cytoplasm. After the viral genome reaches the nucleus, the viral polymerase enzyme will convert the partially double-stranded DNA genome into cccDNA. This is followed by transcription and nuclear export of all viral mRNA to the cytoplasm for translation. The surface protein enveloping process occurs in the endoplasmic reticulum and then assembled in the cytoplasm. These proteins are transported to the post-endoplasmic reticulum and Golgi compartments for the budding of the nucleocapsid. The different viral components will assemble into new virions that will be released out of the host and infect new hepatocyte.

Although HBV genome integration in the host chromosome is not the critical mechanism of HBV pathogenesis, it was found in certain HCC cases that the generated cccDNA persists as a template for the transcription of viral RNA that integrates into the host cell and enables the production of new virions (Figure 3) [68]. Certain host-related factors contribute to these events facilitating HBV persistence, chronicification, and hepatocarcinogenesis. Some of those factors are general circumstances of long exposures to damaging chemicals or oxidative species, repair mechanisms impairment and continuous viral infections [69]. Others include host age during the infection. Early exposure to HBV permits the virus to persist in infants due to immature immune responses and manifest as chronic later in life, unlike in adults. For instance, among chronic HBV-positive patients, 28.8% caught the infection when they were <5 years old, while 7.7% were >30 years old [70]. More specifically, Coursaget et al. reported that HBV-positive infants, <6 months and between 2–3 years, progress to chronic infection in 82% and 15% of the cases, respectively [71]. Consequently, at HBV transmission, the association of age with the duration of chronic infection is inverse.

Additionally, human genes polymorphisms have been reported worldwide to contribute to host susceptibility to chronic HBV and HBV-clearance, of which, IL-4, CTLA4, TLR, TNF, HLA genes, etc. Few studies drew attention to such interest in the MENA region. Starting with TLR, important innate cells receptors, one Tunisian study found significant associations of TLR3 (rs3775290, TT) and TLR4 (rs4986790, GG) minor genotypes with higher risks of chronic HBV-infection [72]. Others found that TLR9 (rs187084, AA) genotype was related to HBV-persistence among Moroccan patients [73]. As for TNF, multi-functional cytokines responsible for cellular activities, TNF-α (rs1800630, −863C/A; rs1800629, −308G/A; rs1800750, −376G/A; rs1800610, +489G/A) and TNF-b1 (T29C) polymorphisms were thought to increase disease susceptibility once seen at high frequencies among Egyptian chronic patients causing decreased cytokine secretion [74,75]. Similarly, TNF-α (rs1800629, −308A; rs361525, −238A) and TNF-R2 variable number tandem repeat (VNTR) (p75) were remarkably linked together to chronic and progressive HBV-infection among Tunisians [76]. Interestingly, one-of-a-kind study in the MENA investigated PD-1 (programmed death-1), a crucial checkpoint inhibitor regulating mainly T-cells and other immune cells, and its possible association with host-response to HBV focusing on rs10204525 multi-genotypes. They found that genotype AA at this allele (rs10204525) was protective against the infection, while GG and GA genotypes increase the risk to develop a chronic infection among Moroccans specifically, and possibly Mediterranean [77]. In regards to HLA genes, encoding for MHC and responsible for immune responses, a Saudi study inspecting the link of HLA polymorphisms to HBV-infection, revealed that variations in HLA-DP (rs3077; rs9277535) and HLA-DQ (rs2856718; rs9275572) were significantly correlated to HBV-infection, mostly in chronic patients [78]. Moreover, a significant association of a single-nucleotide polymorphism (SNP) near FDX-1 (Ferredoxin-1 gene on chromosome 11) with chronic infection was displayed, making this SNP at 11q22.3 a risk allele among Saudi patients [79]. Recently, the same investigators studied the effect of microRNAs variations on host responses. They concluded that, among 10-targeted SNPs in Saudi patients, eight were linked to either, some or all HBV clinical stages (susceptibility, persistence, progression, and clearance). In particular, miR-30a rs1358379 was correlated to all stages. miR-149 rs2292832 and miR-196a2 rs11614913 were correlated to HBV-susceptibility, disease clearance, and progression to HCC/cirrhosis. miR-146a rs2910164 was related to HBV-susceptibility and -clearance and miR-423 rs6505162 was related to viral progression and clearance. miR-492 rs2289030 was uniquely linked to HBV-clearance, while miR-26a1 rs7372209 and miR-608 rs4919510 were uniquely linked to HBV-progression [80]. In comparison to other population-specific SNPs, a review by Akcay et al. collected and summarized worldwide GWAS in an attempt to recognize the role of host polymorphism in HBV-infection, in which HLA topped the list of most related SNPs to HBV pathogenesis and persistence [81]. Overall, researchers in the region are in the right track to identify specific local variations associated with HBV-disease, yet, more studies are needed in this field. Nevertheless, SNPs found amongst indigenous populations in the MENA region need to be confirmed in larger samples, generalized to specific ethnic groups, and integrated into reference databases at the regional and international level.

### 3.2. Viral Factors

#### 3.2.1. Viral Precore (HBeAg) and Core (HBcAg) Mutations

All *hepadnaviruses* share the expression of the pre-core gene product, HBe. This antigen is expressed in infected cells as a modified secreted form of HBcAg [82]. The core protein is highly immunogenic, making it the main target for viral clearance by host immunity. The role of HBcAg is stimulating Th1 immune response, while HBeAg can stimulate both Th1 and Th2 phenotypes to tolerate host immune responses towards HBcAg [83]. Introducing frameshift or point mutations into the pre-core gene could suppress HBeAg expression [84], which is associated with the diminished capability to cause persistent infection [85]. The most common mutation reducing HBeAg levels is a nonsense G1896A, found at pre-core codon region [86], thus preventing HBeAg expression in most cases through stopping pre-core mRNA transcription. Since G1896A nonsense mutation terminates HBeAg, it is frequently found among HbeAg-negative individuals. However, in certain cases, G1896A could also be found in HBeAg-positive individuals in the MENA region. For instance, Ayari et al. found that G1896A mutation was found one out of six Tunisian patients who was HBeAg-positive [87]. Viruses acquire such mutation during persistence to escape host anti-HBe-antibodies, and to create an RNA loop interacting with DNA-polymerase enhancing HBV-replication in each genotype differently [88]. G1896A varies in abundance among HBV genotypes and geographic areas, with genotype A being the least-reported worldwide. In the MENA region, G1896A was reported at high-frequency in Tunisian HBV-patients, mostly in HBeAg-negative compared to HBeAg-positive individuals [87]. Interestingly, the prevalence of this mutation was elevated in genotype E compared to genotype D as previously reported in [87,89]. Likewise, this mutation was reported in UAE and was prevalent in genotype D-carriers [90]. Meanwhile, in Saudi Arabia, precore W28X and G29D were significantly linked to infection progression to cirrhosis/HCC [91].

As for core gene, most common mutations are A1762T and G1764A, which occur in covalently at the promoter. These mutations are reported to affect pre-core RNA transcription, and therefore result in reduced HBe production [92]. Decreased HBeAg expression is not influenced by those mutations only, further positions at the core gene have been linked to this consequence if mutated, such as 1753, 1757, 1766, and 1768 [93]. Furthermore, mutations mentioned above augment genome replication, increasing HBcAg levels by >10-folds compared to wild HBV types via pgRNA up-regulation [93]. In contrast to precore mutations prevalence, core mutations are more common among HBV genotype A, B, and C compared to genotype D [94]. The aforementioned MENA countries reported core mutations in their studies. A1762T/G1764A double mutation was reported among Tunisian population, in HBeAg-positive patients more than -negative ones [87], and in UAE among genotype A and D patients [90]. In Saudi Arabia, >35 core mutations were examined, and only six were significantly correlated to HBV progression (i.e., F24Y, E64D, E77Q, A80I/T/V, L116I, and E180A), through changing specific HBcAg immunogenic epitopes to escape B-cells and T-cells neutralization [91].

#### 3.2.2. Viral PreS/S (HBsAg) Mutations

HBsAg, an expressed protein on the surface of the virus, is one of the early viral markers for HBV active or acute infection [95]. HBsAg level in the serum is associated with cccDNA levels inside host hepatic cells, defining a clinical relevance of this marker [96]. For that, HBV-infection can be indirectly assessed by quantifying levels of circulating HBsAg to determine the infection history, status of HBV-infected patients (e.g., inactive carriers, chronic, or reactive patients) and treatment outcomes [88]. Similar to HBcAg, HBsAg is highly immunogenic and able to trigger the activation of CD8+ T-cells by dendritic cells (DCs) and macrophages antigen presentation without inflammatory cytokines involvement [97]. In some chronic patients though, HBsAg can inhibit cytokine production of DCs and macrophages through Toll-like receptors (TLR) signalling and can manipulate innate responses [98]. While in other chronic patients, HBsAg can trigger HBV-specific T-cells through antigen presentation of macrophages [99]. Introducing deletion or point mutations at certain PreS regions have been linked to HCC development [100]. Whether located in C- or/and N-terminus regions of PreS1 or/and PreS2, the main consequence of those mutations is the production of short forms of HBsAg, affecting T-cells and/or B-cells recognition sites and thus escaping adaptive immune system [82]. Moreover, mutant full-length HBsAg can induce oxidative stress and replication impairment of host cells, degrade a cyclin-dependent kinase inhibitor (cell-cycle inhibitor) p27 and trigger cell transformation [101]. Most prevalent genotypes with mutant HBs are mainly genotypes C and B, isolated from chronic patients and related to chronic infection and HCC formation [102]. In MENA region, P127S/T, an escape mutation, P120T, responsible for low HBsAg expression in-vitro, Y134F and S143L mutations were detected in chronic HBV Egyptian patients and reported mostly in genotypes B and D (subgenotype D3/D7) [103,104]. F130L and S135F mutations were found in Saudi patients carrying genotype D (subgenotypes D1/D3), with unknown effect to HBsAg and unknown association to the disease pathogenesis [105]. In Palestine, reported mutations in this gene were T126T, P127S, G145R, and D144E escape mutations among HBV genotype D (subgenotype D1) HBsAg-positive patients [66]. Lastly, in Tunisia, the most abundant mutation out of many detected ones was S143L/T, which was prevalent among genotype D (subgenotype D7) carriers and significantly related to viral progression to cirrhosis. Most substitutions belonged to T-helper, T-cytotoxic, and B-cells epitopes, in an attempt to decrease viral–host binding affinity [106].

#### 3.2.3. Viral X Gene (HBxAg) Mutations

HBx gene is a small gene producing a short conserved protein among all hepadnaviruses [107]. Its main functions are dormant cell activation and HBV promoters’ stimulation through transcription factors or signaling pathways [107]. HBx has been connected to cell apoptosis through Ca^2+^ signaling causing elevated oxidative stress [108]. Moreover, the latter can ensure infected liver cells survival, through blocking TNF-α and activating NF-kB, promoting persistent HBV-infection [109] and consequently HCC formation through PYK2 and SRC pathways [110]. These findings might clarify the relationship of HBx to severe chronic liver damage. Introducing mutations at certain *x* gene regions and overlapping with core gene at 1753, 1762, 1764, and 1768 positions, result in C-terminal alteration of HBxAg, which inhibit cell replication to an extent and p21 expression [111]. In MENA countries, only Saudi Arabia reported mutations in this gene, such as I127T, V131I, F132Y/I/R, H94Y, and K130M mutations. These variations were significantly linked to severe infection and progression to HCC. Once K130M+V131I, with/without a third mutation, were found together the risk of developing HCC increased more compared to single mutation cases [112]. One year later, similar investigators conducted functional analysis research for some of these mutations along with other deletion mutations. They found that full-length (1–154) and short-length versions (1–94; 31–152; 61–154; 61–124) of HBx were affecting cell cycle through p53 inhibition by truncated forms and through p27, p21, and cyclin D1 overexpression by complete form. Notably, C-terminal and N-terminal deletions of HBx were in favour of the protein’s oncogenic property, and in favour of check-point inhibitor overexpression, respectively [113].

In Summary, further mutational analysis is needed to reveal the role of reported mutations and the affected pathways. Moreover, deeper inspection of mutant viruses circulating in MENA countries in correlation to their genotypes’ prevalence and the clinical disease stages.

## 4. HBV Coinfections

Coinfection of hepatitis B patients with HCV, human immunodeficiency virus (HIV), hepatitis E virus (HEV), torque teno virus (TTV) and human pegivirus (HPgV), formerly known as GB virus C/hepatitis G virus (GBV-C/HGV), has already been reported [114,115]. However, prevalence, viral interactions and clinical significance of such coinfections are yet to be fully elucidated, particularly in the MENA region. McArdle et al. [116] stated that viral coinfections could give rise to insignificant, deleterious or beneficial effects in patients by altering normal host response, changing diagnostic tests performance, and modulating host responses to treatment [116]. These possibilities will be further explored in the context of HBV coinfection with each of HCV, HIV, HEV, TTV and HPgV (GBV-C/HGV).

### 4.1. HCV/HBV Coinfections

As previously mentioned, OBI refers to HBV infections that are “serologically silent”. This is characterized by having low levels of serum and/or liver HBV DNA with undetectable serum HBsAg [117]. This virologic phase is seen in HBV-patients with HCV superinfection in Mediterranean Basin and Far East Asian countries with a prevalence of 33% and 50%, respectively [118]. Some studies argue that HCV superinfections suppress HBV replication in patients with HCV/HBV coinfection [117], resulting in undetectable serum HBsAg levels seen in occult HBV virologic phase. Furthermore, in HCV/HBV coinfected-patients with occult HBV, clearance of HCV using pegylated interferon-α (peg-IFN-α) and ribavirin resulted in HBV-reactivation, further supporting the hypothesis that HBV replication is suppressed by HCV [119]. However, since occult HBV is not always accompanied by HCV coinfection, Fukuda et al. argue that other factors, such as the 8-nt mutation in HBV promoter, could be responsible for reduced HBV replication seen in occult HBV [120]. On the other hand, an HCV/HBV coinfection is deemed clinically significant as it results in higher morbidity rates than a single HBV infection. Fong et al. reported that higher rates of liver cirrhosis are observed in coinfected patients as compared to those with a single HBV-infection (44% vs. 21%) [121]. The same study also reported an increased prevalence of the decompensated liver disease in patients with dual infection (24% vs. 6%). Similarly, one longitudinal study shows that the cumulative risk of developing HCC is higher in patients with a double infection than in patients with an HBV mono-infection (45% vs. 16%) [122]. Therefore, Donato et al. suggested a synergistic interaction between both viruses in causing HCC where HBV initiates the neoplastic process, and HCV consequently acts as a promoter, thus worsening the liver status of coinfected individuals [123].

### 4.2. HIV/HBV Coinfections

HIV/HBV coinfections are by far one of the most explored coinfections due to their high prevalence. This is mostly attributed to the viruses having shared modes of transmission, i.e., sexual and percutaneous routes. In HIV-infected populations, HBV coinfection rates could reach 25% in countries where the viruses are endemic, and >10% in Northern Asia and other countries where HBV is not prevalent [124]. This rate varies according to geographical locations and modes of acquiring the infection. For instance, HBV/HIV infections were found to not exceed 1.5% in Egypt, KSA, and Turkey [125,126,127]. The mode of transmission also appears to play a role where men who have sex with men (MSM) were found to have the highest rates of coinfection as compared to heterosexuals and injecting drug users (IDUs) [124]. Considering that HBV is around 100-times more likely to be acquired than HIV, HBV infections typically precede HIV infections [124].

The clinical challenges imposed by this coinfection is found to be similar to those of an HCV/HBV coinfection where dual infections result in higher liver-related mortalities than single HBV infections [128]. Studies showed that patients with dual infection were significantly more probable to be HBeAg sero-reactive as compared to patients with HBV mono-infection (37% vs. 10%). These patients were also found to have higher HBV viremia (≥4.3 log IU/mL) than mono-infected individuals (37% vs. 16%) [129]. Studies by Kourtis et al. found that progression to chronic hepatitis B is around five-fold quicker in coinfected individuals as compared to HBV mono-infected individuals [130].

### 4.3. TTV/HBV Coinfections

Throughout the MENA region, HBV-coinfection with TTV appears to be a common occurrence with percentages ranging from 31.3% [131] in Turkey to 90.7% [132] and 88.8% [133] in Qatar and KSA, respectively. When the effect of this coinfection was evaluated through the measurement of serum ALT levels, studies found no significant TTV-induced biochemical response in coinfected individuals [115,133]. Studies carried out in Egypt also found the prevalence of HCC and liver cirrhosis in patients with the HBV/TTV coinfection to be of no statistical significance when compared with groups without the coinfection [134]. On the contrary, the histological liver investigation of 25 pediatric patients with chronic hepatitis B revealed that those with TTV viremia had a significantly higher histologic activity index, periportal necrosis, as well as portal inflammation scores than those with a simple HBV infection. Kasirga et al. also reported that HBV infected individuals are significantly more likely to harbour a TTV infection suggesting a possible shared route of transmission between the two viruses [135]. This finding contrast with the findings from our lab, we reported no significant difference between the two groups mentioned earlier [132]. The relatively high omnipresence of TTV in healthy blood donors, particularly in the MENA region, raises questions regarding the clinical and pathologic significance of an HBV/TTV coinfection [132,136].

### 4.4. HEV/HBV Coinfection

The prevalence rate of HEV/HBV coinfections appears to vary greatly in the MENA region with rates ranging from 0% to 56% [137,138]. Although HEV can be transmitted both vertically and through blood transfusions, the virus is mainly transmitted through the faecal–oral route due to poor sanitation conditions [139]. This variation in transmission modalities could account for the differences in the rates of coinfections in the MENA region. Since HEV is a causative agent of acute and chronic liver inflammation [140], it is expected for an HEV/HBV coinfection to have significant clinical outcomes. However, a clear link between these two viral infections is yet to be established [141], Studies done by Hoan et al. reported the seroprevalence of HEV to be significantly higher in hepatitis B patients than in controls (45% vs. 32%, P = 0.034) [142]. The same group also found coinfected patients with liver cirrhosis to have a higher anti-HEV IgM prevalence than patients without liver cirrhosis (16.8% vs. 9.5% P = 0.01) suggesting a correlation between an HEV superinfection and liver cirrhosis in HBV infected individuals [142].

### 4.5. HPgV/HBV Coinfection

The prevalence of HPgV infection in HBV infected patients around the MENA region are relatively comparable with rates of 4.1%, 7.8%, 10.7%, and 12% in KSA, Turkey, Qatar, and Iran respectively [131,136,143,144]. Studies conducted by Fiordalisi et al. [145] and Yoshiba et al. [146] reported HPgV to be associated with non-A-E hepatitis. In contrast, a study from our lab reported that there is no correlation between HBV and HPgV infection [136]. Similarly, Laskus et al. [147] and Alter et al. [148] reported that the virus does not appear to be hepatotropic as it does not replicate in hepatocytes nor was it found to cause chronic or acute hepatitis. This is in concordance with studies investigating the effect of an HPgV and HBV coinfection on the liver where the mean ALT level of patients with the coinfection was significantly lower than those with a single HBV infection [149]. Studies by Kao et al. showed that HPgV superinfection in HBV infected patients did not cause a significant change in HBV DNA levels unlike HCV superinfection [150]. HPgV hence does not seem to mimic the synergistic interaction of HCV/HBV coinfections. Therefore, HPgV superinfection might not worsen disease status, at least not in the context of the liver. However, data-addressing HPgV in HBV infected patients is scarce, and so the clinical significance of the coinfection remains poorly understood.

## 5. Laboratory Diagnosis of HBV

**Figure 4:**
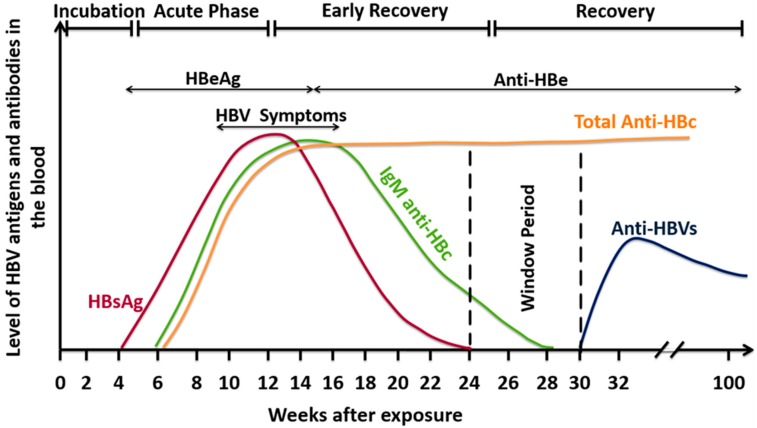
A graph is illustrating the pathogenic events throughout HBV infection. HBsAg can be detected very early in the acute course of infection and starts declining in serum to undetectable levels within 23–24 weeks post infection. The HbeAg is next and indicates the ability to infect others. The first HBV antibody produced is HBc IgM, and it may persist until 28 months post infection. Hence, detection of IgM represents an acute HBV infection. However, in the chronic infection phase, IgG becomes detectable and persists for a more extended period than IgM. During the recovery period, anti-HBs will not appear for a few weeks after HBsAg has been cleared. It is possible for both HBsAg and anti-HBs to be negative during recovery. This is called the window period in acute infection. Later, anti-HBs will be developed, and the immune system develops immunity as a result of an actual infection.

HBV infection is considered a significant global health threat. Accurate diagnosis of HBV infection is crucial for treatment. There are several stages of HBV infection, where each is characterized by the presence of specific biomarkers such as viral DNA or human antibodies against viral antigens (Figure 4). Therefore, detection techniques are classified into serological assays with different sensitivities and specificities (Table 2) as well as molecular assays for HBV DNA detection using different forms of PCR. These tests might help recognize the onset and stage of HBV infection. Indeed, if both serological and molecular techniques are combined, they will add substantial value toward an accurate diagnosis of HBV infection.

HBsAg is the first detectable marker in serum during the acute infection [95], and its prevalence in the MENA region has been summarized in Table 1. Laboratory diagnosis of HBV infection in the MENA region has been mostly based on HBsAg detection. However, since the HBsAg level could be lower than the detection limit, or present with mutations in the antigen epitopes, molecular techniques have been considered as an important tool for efficient and accurate HBV detection in several countries in MENA regions. The molecular methods are useful in quantifying the viral load, genotyping, and detecting drug resistance mutations [151]. Molecular techniques include amplifying of HBV DNA using thermal cycling-based techniques such as polymerase chain reaction (PCR), transcription mediated amplification (TMA), loop-mediated isothermal amplification (LAMP), and rolling circle amplification (RCA), etc. Each method has advantages and disadvantages that promote or limit their use in clinical diagnosis.

Due to slight modifications of conventional PCR in detecting the desired gene sequence, several forms of PCR have been introduced [140]. Since automated real-time PCR has a high capacity to detect a broad range of viral load and lack carry-over contamination, it is considered the standard method for detecting and quantifying HBV DNA [151]. Other types of PCR that have been used for HBV diagnosis include multiplex and droplet digital PCR (ddPCR). ddPCR is one example of modern molecular testing, and it was found to be a sensitive method to detect cccDNA in HBV samples. The HBV genome is a partially double-stranded DNA that encodes four overlapping genes (Figure 1). The serum HBsAg can be quantified by molecular assays such as the Elecsys HBsAg II Quant (Roche Diagnostics, Penzberg, Germany) [155] and Architect HBsAg QT (Abbott Laboratories, Rungis, France) [156]. The advantages of these assays are their ability to detect all forms of circulating HBsAg as well as they are fully automated with a high throughput capacity as well as low cost. PCR based methods are the most practised techniques, yet, other methods such as TMA, LAMP, and RCA have been employed to detect and quantify HBV DNA. However, false-positive results and contamination are the major drawbacks of the amplification-based assays, which can be avoided by following precautions as well as the use of proper controls. Another molecular diagnostic method is genotyping by direct sequencing of HBV DNA, which is useful for studying viral mutations and genotypes. However, this technique is not adapted to high-throughput screening and is found to be labour-intensive, and time-consuming. Combining both serological and molecular methods would improve the early detection of the virus and diagnose the infection more accurately [140].

## 6. Conclusions

HBV is a highly prevalent virus where 257 million people are living with this infection worldwide. In the Gulf region, HBV infection rates reached up to 20% and it is considered the second most common causative agent of HCC following HCV. Genetic variability in the HBV genome resulted in ten genotypes distributed across different geographical regions worldwide, where genotype D is the most abundant HBV genotype in the MENA region. HBV pathogenesis and severity of infection depend on several host and viral factors, particularly, the genetic variability of both, the host and virus as discussed previously. Although the symptoms of HBV infection are not distinguishable from other hepatitis infections, there are different clinical stages characterized by the presence of specific biomarkers. Therefore, combining both serological and molecular methods would improve the early detection and accurate diagnose of HBV. The incidence and prevalence of HBV infection have sharply declined in the MENA region due to the implementation of safety measures and effective universal vaccination programs. Still, the transmission of HBV in some developing countries remains a significant risk because of the limited access to personal protective equipment and HBV vaccination.

## Figures and Tables

**Table 1:** Prevalence of hepatitis B surface antigen (HBsAg) among people in the Middle East and North Africa (MENA) region using different detection methods.

Country	Sample Size	Prevalence (%)	Diagnostic Assay Used	Year	Reference
**Turkey**	101,648	4	Elecysys HBsAg II ELISA (Roche Diagnostics, Germany)	2018	[6]
	1404	6.6	micro-ELISA method	2003	[7]
	12,010	3.8	ELISA	2013	[8]
	30,716	2.2	-	2011	[9]
	10,391	8.1	ELISA E170 (Roche, Germany)	2010	[10]
**Iran**	6583	2.6	Enzygnost HBsAg 5.0 kit (Dade Behring, Germany)	2009	[11]
	708	0.28	Radim kit (KHB31WB) through immunoenzymometric assay	2010	[12]
	284	0.35	ELISA using the EIAgen HBsAg Kit	2011	[13]
	124,704	0.24	ELISA	2014	[14]
	20,591	0.23	The DIASORIN (Italy) kits	2012	[15]
	2,026,628	0.38	Third generation ELISA kits	2014	[16]
**Pakistan**	7000	2.5	ELISA Abbott Determine (TM)	2010	[17]
	11,900	9.8	Immunochromatography technique-based kit commercially (Determine-Abbott USA).	2011	[18]
	127,828	2.68	BEST 2000 ELISA (Biokit, Spain)	2012	[19]
	2155	1.34	-	2013	[20]
	160,376	2.35	Fourth generation ELISA kits (Bio-kit)	2014	[21]
**Afghanistan**	330	3.6	Fast cassette kits	2014	[22]
**Palestine**	146	8.2	ELISA	2014	[23]
	17,060	3.8	Abbot EIA	2002	[24]
	399	2.8	ELISA	2004	[25]
**Jordan**	62,933	0.52	Murex HBsAg Version 3 ELISA kit (DiaSorin S.p.A., Dartford, UK) or BioRad Monalisa HBsAg sandwich ELISA kit (Bio-Rad, Marnesla Coquette, France).	2016	[26]
**Lebanon**	16,084	0.92	Hepanostika HBsAg Uni-Form II, a sandwich ELISA (Biomerieux, Marcy l’Etoile, France)	2006	[27]
**Yemen**	521	16.9	Monolisa enzyme immune assays (BIO-RAD, France)	2012	[28]
	3000	2.1	ELISA	2014	[29]
	400	10.8	Fourth generation ELISA	2013	[30]
**Iraq**	9610	1.6	ELISA	2013	[31]
	23,336	0.73	ELISA	2010	[32]
	495,648	0.66	ELISA	2011	[33]
**Qatar**	78,428	0.9	-	2007	[34]
	495	1.0	ELISA (Axsym, Abbott Laboratories)	2006	[35]
**Oman**	604	7.1			
	
**UAE**	595	1.5			
**Saudi Arabia**	10,234	5.9	Fourth generation ELISA	2017	[36]
	8501	0.7	chemiluminescent microparticle immunoassays (ARCHITECT^®^ HBsAg, ARCHITECT^®^)	2016	[37]
	2807	0.8	ELISA (BioRad, Marnes–la-Coquette, France)	2016	[38]
	29,949	3.8	ELISA (Siemens-BEPIII, Dade Behring, Marburg, Germany)	2013	[39]
	3192	3	EIA (Abbott Laboratories, Chicago, IL, USA)	2008	[40]
	2330	11.8	PCR and agarose gel electrophoresis	2004	[41]
**Bahrain**	7714	3.7	EIA/ELISA, and reconfirmed by PCR	2004	[41]
**Kuwait**	12,798	1.92	-Chemiluminescent immunoassays -Neutralization confirmatory assay (Auszyme monoclonal, Abbott Laboratories, Abbott Park, IL)	2005	[42]
**Egypt**	12,000	1.98	ELISA	2013	[43]
	308,762	1.22	Enzygnost HBsAg 6.0	2014	[44]
**Sudan**	5965	9.76	-	2015	[45]
	178	21.3	ELISA	2016	[46]
	404	11	Enzygnost HBsAg; 5.0 EIA	2011	
**Morocco**	23,578	1.81	Murex HBsAg Version 3	2013	[47]
**Libya**	65,761	2.2	Third generation microparticle EIA (Axsym)	2013	[48]
	1500	1.5	ELISA	2010	[49]

**Table 2:** Sensitivity and specificity of different commercial immunoassays mostly used for HBV detection.

Company name	Coating	Biomarker detected	Sensitivity (%)	Specificity (%)	Sample size	Reference
Elecsys^®^ HBsAg II assay	Anti-HBsAg	HBsAg	100%	99.97%	9084	[152]
Architect	-	HBcAb	79.90%	98%	260	[153]
	-	HBsAg and HBcAb	100%	90%		
Elecsys	-	HBcAb	78.90%	90.20%		
Hepalisa	Direct sandwich ELISA, microwell plates coated with Anti- HBsAg	HBsAg	100%	100%	100	[154]
Microscreen HBsAg	Microwell plates coated with Anti- HBsAg		100%	97.8%		
ERBA LISA HEPATITIS B	Sandwich ELISA, microwell plates coated with Anti- HBsAg		100%	100%		
HEPACARD	Immunochromatographic assay, membrane coated with Anti-HBsAg	HBsAg	100%	100%	100	[154]
Crystal HBsAg						
SD BIOLINE HBsAg						

## Abbreviations

ALT	Alanine Aminotransferase
cccDNA	Covalently closed circular DNA
CDC	Centers for Disease Control and Prevention
DCs	Dendritic cells
ddPCR	Droplet digital PCR
GWAS	Genome-wide association studies
HBc	Hepatitis B core protein
HBcAg	Hepatitis B core antigen
HBeAg	Hepatitis B e antigen
HBeAb	Hepatitis B e antibody
HBsAg	Hepatitis B surface antigen
HBsAb	Hepatitis B surface antibody
HBV	Hepatitis B Virus
HCC	Hepatocellular carcinoma
HCV	Hepatitis C Virus
HEV	Hepatitis E Virus
HIV	Human Immunodeficiency Virus
HPgV (GBV-C/HGV)	Human Pegivirus (GB virus C/Hepatitis G virus)
IgM	Immunoglobulin M
IgG	Immunoglobulin G
MENA	Middle East and North Africa
OBI	Occult hepatitis B virus infection
PCR	Polymerase chain reaction
PD-1	programmed death-1
Th1	T-helper 1 cells
Th2	T-helper 2 cells
TLR	Toll-like receptors
TTV	Torque Teno Virus
WHO	World Health Organization
